# What’s Inside of a AA Battery? An Unusual Caustic Ingestion in an Infant

**DOI:** 10.1097/PG9.0000000000000118

**Published:** 2021-08-26

**Authors:** Harrison M. Luttrell, William E. Bennett, Paroma Bose

**Affiliations:** From the * Department of Pediatrics, Indiana University School of Medicine, Riley Hospital for Children, Indianapolis, IN; † Department of Pediatrics, Division of Pediatric Gastroenterology, Hepatology, and Nutrition, Indiana University School of Medicine, Riley Hospital for Children, Indianapolis, IN

**Keywords:** esophageal burn, corrosive injury, alkaline battery, cylindrical battery

## Abstract

Current guidelines for the management of battery ingestions in children focus on button batteries due to the risk of morbidity and mortality. In our review of the literature, there is little information on the ingestion of cylindrical AA or AAA battery contents. We report a case of an 11-month-old female who ingested the internal alkaline contents of a AA battery. The ingestion resulted in oropharyngeal and esophageal caustic injuries visualized on upper endoscopy. Imaging has long been used for localizing ingested whole batteries. In our case, standard radiograph confirmed that internal battery contents were ingested. Advanced imaging modalities, including computed tomography, have been suggested as methods to investigate the degree of caustic injury and were utilized in this case. Our case is one of the few reported cases of the ingestion of alkaline battery contents alone.

## INTRODUCTION

With the continued integration of technology in our society, there has been increasing incidence of injuries from battery ingestions in the United States (1). Current guidelines for the management of battery ingestions in children focus on button batteries (2,3), due to the risk of morbidity and mortality (1). There is limited guidance regarding management and outcomes of cylindrical battery ingestions, which carry risk of both electrothermal and caustic injury. We present a case of an 11-month-old child who suffered esophageal caustic injury after ingesting the internal contents of a cylindrical alkaline battery.

## CASE REPORT

**FIGURE 1.**
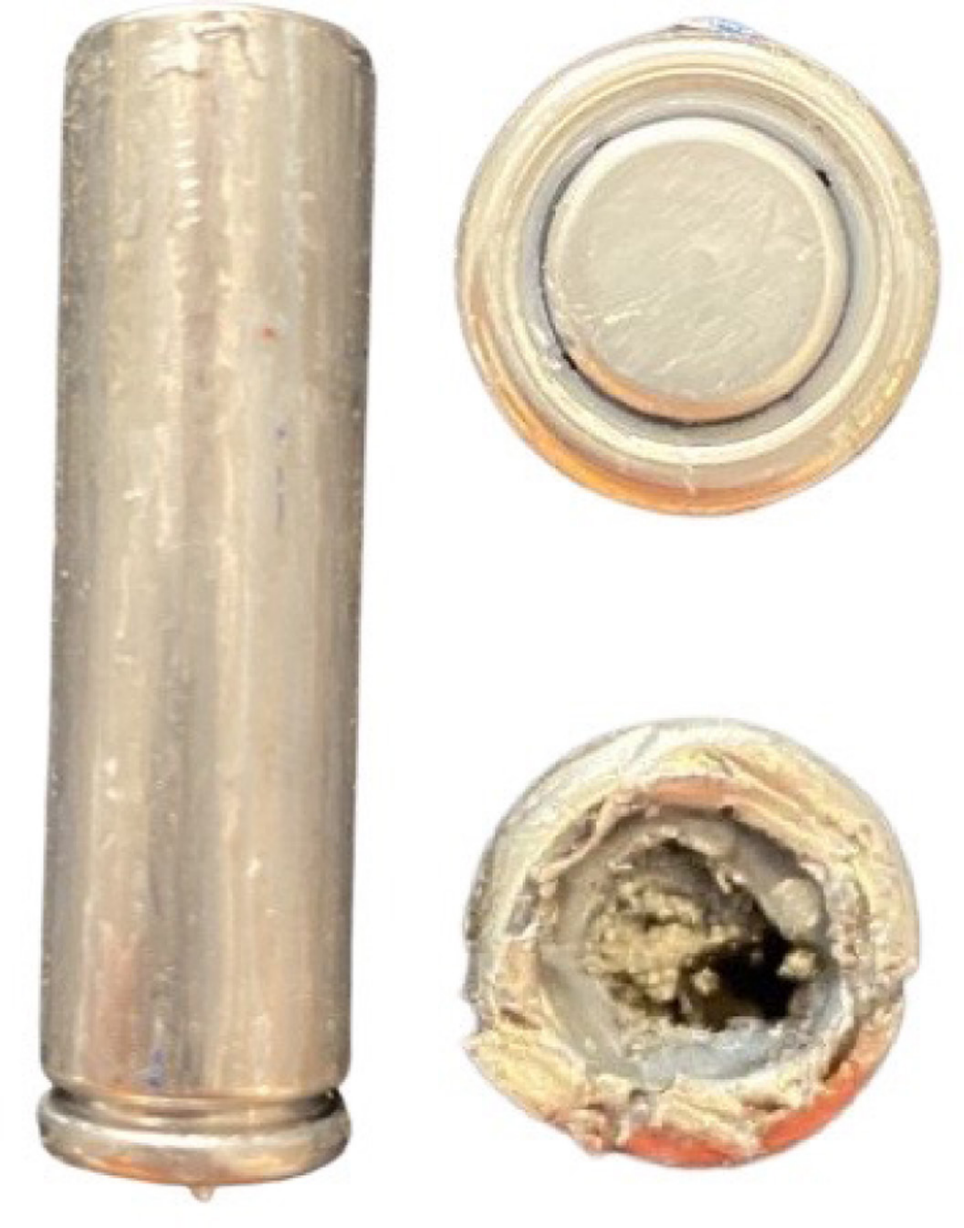
Representative cylindrical AA battery with negative electrode removed and visible internal contents.

The 11-month-old female was playing alone in the living room when her mother heard a loud “pop.” She found the patient crying and with mild swelling of her lower face and tongue. Nearby was a “CSE Alkaline” AA battery with a deformity where the negative electrode had been removed (Fig. 1). The internal contents of the battery were no longer present. The negative electrode was later found separate but intact. Her mouth and face were immediately washed with water. She was transported to a local hospital by ambulance.

**FIGURE 2.**
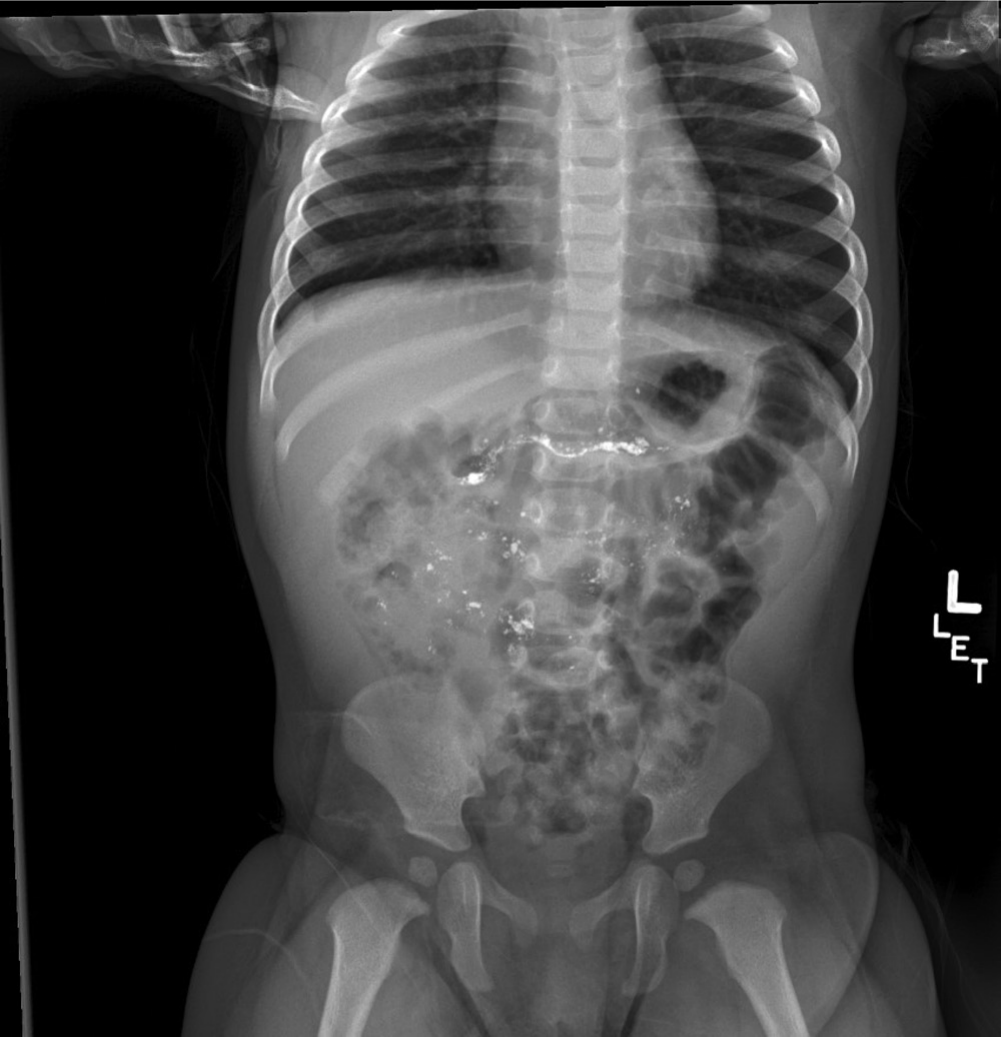
Abdominal radiograph approximately 2 hours after ingestion demonstrating hyperdense material in the proximal small intestine.

**FIGURE 3.**
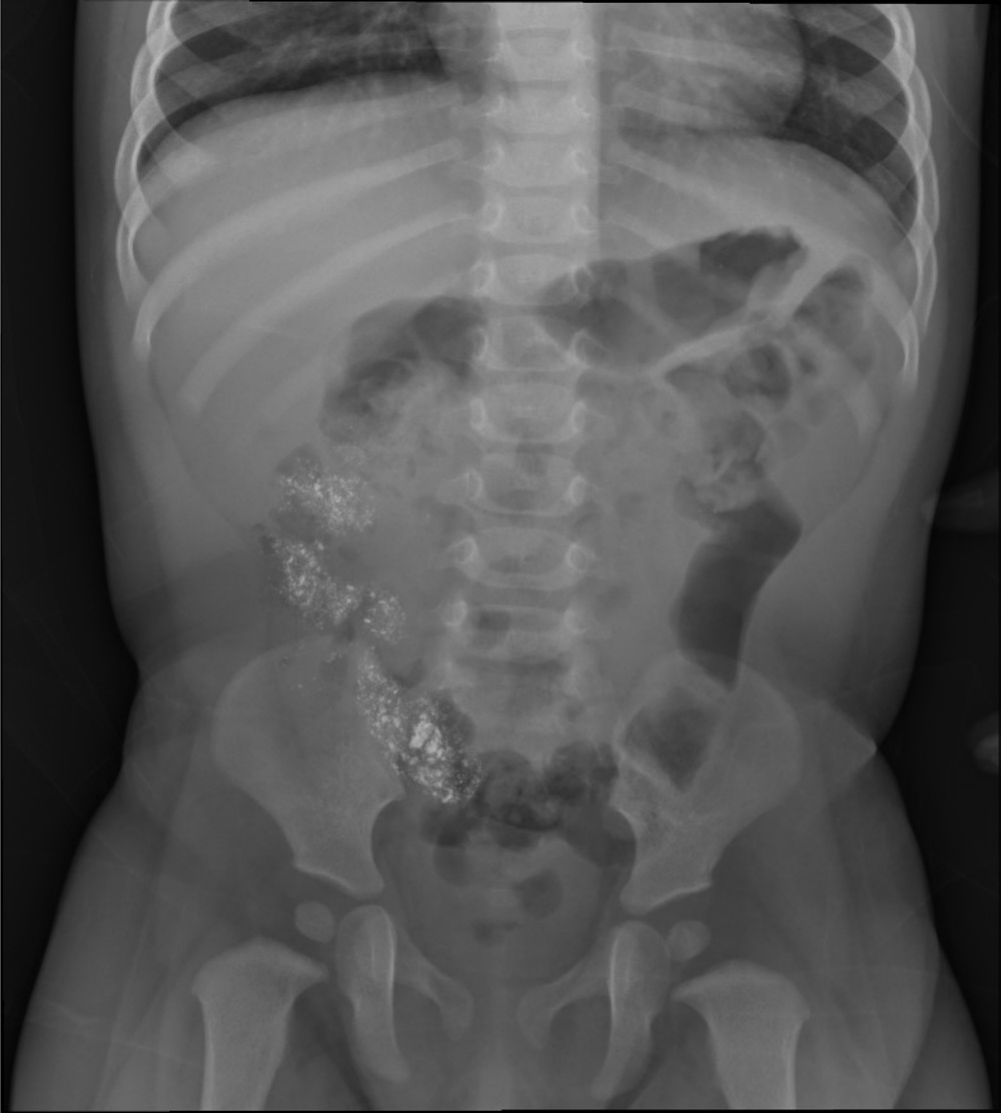
Abdominal radiograph approximately 7 hours after ingestion demonstrating hyperdense material in the ascending colon.

Upon initial evaluation, radiograph of the chest and abdomen showed hyperdense material in the small intestine (Fig. 2). On arrival to our tertiary-care pediatric hospital thereafter, she was refusing to swallow her oral secretions. White fibrinous debris was seen over the hard palate and dorsal surface of the tongue without posterior oropharyngeal lesions. She had mild erythema overlying her left cheek just below her eye without conjunctival injection or periorbital swelling. Repeat abdominal and chest radiographs showed dense material over the cecum and ascending colon (Fig. 3). The local Poison Control Center was contacted for the first time at our hospital and provided their caustic ingestion protocol and contact information for the National Battery Ingestion Hotline, with whom a case was filed. Per established clinical guidelines, pediatric gastroenterology recommended a proton-pump inhibitor and inpatient admission for esophagogastroduodenoscopy (EGD) within 24 hours (2,4).

**FIGURE 4.**
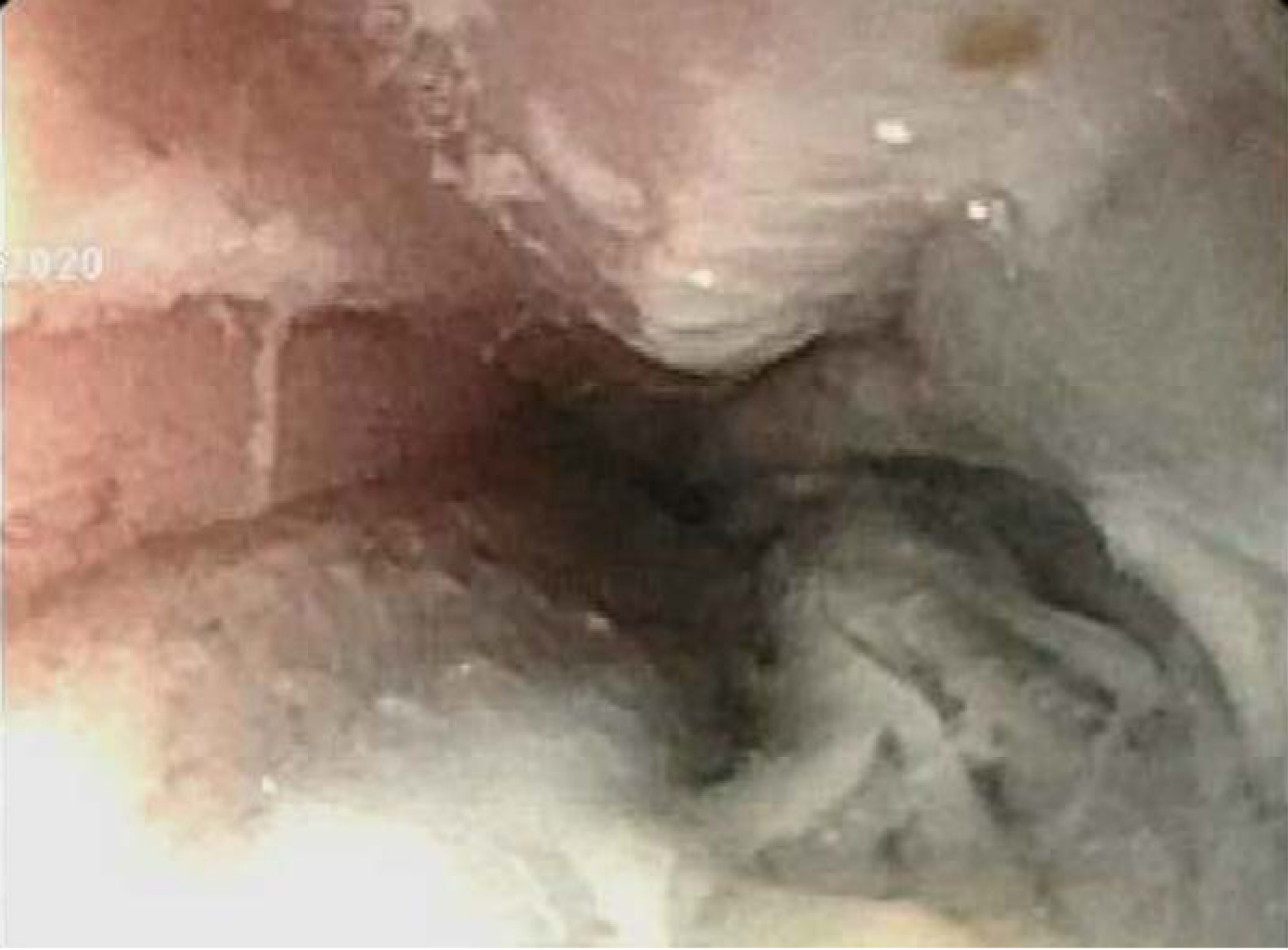
EGD image showing grade IIb-III esophageal injury. EGD = esophagogastroduodenoscopy.

Approximately 14 hours after the ingestion, EGD showed extensive ulceration and adherent fibrin in the lower esophagus. There was an area above the lower esophageal sphincter with a dark appearance indicative of recent bleeding or necrotic tissue, consistent with grade IIb–III injury (Fig. 4). A nasogastric (NG) tube was placed endoscopically. A radiograph was done to assess placement and incidentally demonstrated a new left upper lobe opacification. A subsequent computed tomography scan of the chest without contrast showed wall thickening of the distal esophagus without evidence of perforation or pneumonitis. She was fed via NG tube for 2.5 days, after which oral feedings were reintroduced. Due to the concerns for aspiration, she was empirically treated with ampicillin/sulbactam followed by amoxicillin/clavulanate for a 7-day course. A repeat abdominal radiograph on day 3 postingestion showed further transit of the radiopaque material throughout the colon and rectum. During her hospitalization, she was followed by the inpatient toxicology team and the Poison Control Center. Ophthalmology did not find any caustic ocular injury. She was discharged home on hospital day 4 on an oral diet with plans for a barium esophagram and repeat EGD in 6 weeks. Despite parental report of tolerating oral feeds a week after discharge, she has since been lost to follow up.

## DISCUSSION

Rather than a button battery or an entire battery capsule, our case involves the ingestion of only the internal liquid contents from a cylindrical AA battery. In an alkaline AA battery, the internal contents are a mixture of zinc-manganese dioxide and sodium or potassium hydroxide (5). When exposed to tissue, these alkaline contents can cause liquefactive necrosis (6). To our knowledge, this is one of a few reported cases of sole ingestion of the internal contents of an alkaline battery in a child. We found one other case of an esophageal burn in a 4-year-old patient ingesting alkaline battery liquid by sucking on the casing of a leaky battery, which resulted in grade II–III injury and recurrent esophageal stenoses (7).

Considering the internal contents of a AA battery, we treated our case as a caustic ingestion and performed endoscopy within 24 hours. This management strategy is consistent with that recommended by the European Society for Paediatric Gastroenterology, Hepatology and Nutrition (ESPGHAN) guidelines for corrosive ingestions (4). Our team also considered the possibility of esophageal edema and feeding difficulty and pre-emptively placed a nasogastric tube.

Based on recent literature, button battery ingestion guidelines now advocate for the use of honey or sucralfate soon after ingestion to minimize the severity of injury and risk of perforation (3,8). These agents neutralize pH and create a physical barrier between esophageal mucosa and the button battery, preventing closure of the electrical circuit that leads to injury. It is unclear whether we can expect similar outcomes in cases like ours, which involve caustic injury directly from an alkaline substance.

Imaging in our case demonstrated a progression of the battery contents through the gastrointestinal tract. There are cases in the literature reporting gastric and colonic injury after intestinal ingestion of caustic materials (9). However, in our case, EGD did not show mucosal injury beyond the distal esophagus, and our patient had no clinical signs of further gastrointestinal involvement. To our knowledge, there are no recommendations or guidelines that advocate for colonoscopy after caustic ingestion.

This case is a reminder that some cylindrical batteries contain caustic alkaline fluid. Management of an alkaline cylindrical battery ingestion therefore should address both the risks of caustic injury from the liquid contents and electrothermal injury if the battery is ingested intact. Ingestions of a leaking battery or battery contents need only be managed as a caustic ingestion. We advocate for early endoscopic evaluation based on caustic ingestion guidelines (2,4) to assess for alkaline-induced mucosal injury. A multidisciplinary and interprofessional approach may be necessary depending on affected structures. Although devastating complications from button battery ingestions are well documented (1), this case suggests that children who ingest the internal liquid battery contents may have good outcomes. Further consideration of the ideal management of these patients is certainly warranted.
